# Rapid glycolytic activation accompanying innate immune responses: mechanisms and function

**DOI:** 10.3389/fimmu.2023.1180488

**Published:** 2023-04-20

**Authors:** Dmitry Namgaladze, Bernhard Brüne

**Affiliations:** ^1^ Institute of Biochemistry I, Faculty of Medicine, Goethe-University Frankfurt, Frankfurt, Germany; ^2^ Fraunhofer Institute for Translational Medicine and Pharmacology ITMP, Frankfurt, Germany; ^3^ German Cancer Consortium (DKTK), Partner Site Frankfurt, Frankfurt, Germany; ^4^ Frankfurt Cancer Institute, Goethe-University Frankfurt, Frankfurt, Germany

**Keywords:** macrophage, dendritic cell, metabolism, glycolysis, inflammation, pattern recognition receptors

## Abstract

Innate immune responses to pathogens, mediated by activation of pattern recognition receptors and downstream signal transduction cascades, trigger rapid transcriptional and epigenetic changes to support increased expression of pro-inflammatory cytokines and other effector molecules. Innate immune cells also rapidly rewire their metabolism. The most prominent metabolic alteration following innate immune activation is rapid up-regulation of glycolysis. In this mini-review, we summarize recent advances regarding the mechanisms of rapid glycolytic activation in innate immune cells, highlighting the relevant signaling components. We also discuss the impact of glycolytic activation on inflammatory responses, including the recently elucidated links of metabolism and epigenetics. Finally, we highlight unresolved mechanistic details of glycolytic activation and possible avenues of future research in this area.

## 1 Introduction

Pathogen sensing by a variety of cell surface and intracellular pattern recognition receptors (PRRs) initiates an immediate response of innate immune cells aimed at eliminating the danger. PRRs activate well-established intracellular signaling cascades that culminate in activation of transcription factors of the NFκB, AP-1, and IRF families. In turn, this increases the transcription and protein synthesis of pro-inflammatory cytokines, chemokines, and other molecules associated with the inflammatory response (1). In addition, PRR activation induces substantial alterations of cellular metabolism, most prominent being activation of glycolysis. As the inflammatory response progresses, dynamic remodeling of innate immune cell metabolism occurs (2), often as a result of expression changes of multiple metabolic enzymes. This remodeling involves not only upregulation of the glycolytic flux, but also changes in the activities of pentose phosphate pathway (PPP), tricarboxylic acid (TCA) cycle, mitochondrial respiratory chain, fatty acid and amino acid metabolism (3–5). In most of these cases, metabolic alterations are described to accompany pro-inflammatory activation occurring within hours after PRR activation. The multiple changes are often associated with a transition of the cell towards a state less responsive, or tolerant to a recurrent stimulation (2). Initial responses to pathogens, however, occur on a time scale of minutes, and include both transcriptional activation (6) and up-regulation of glycolysis (7, 8). In this mini-review, we will discuss the possible mechanisms linking PRR-induced signaling to glycolytic activation and elaborate the impact of early changes of cellular metabolism on pro-inflammatory responses. For more general information on metabolic reprogramming during innate immune responses, the reader is referred to several comprehensive reviews (9–12).

## 2 Mechanisms linking pathogen sensing to glycolytic activation

**Figure 1:**
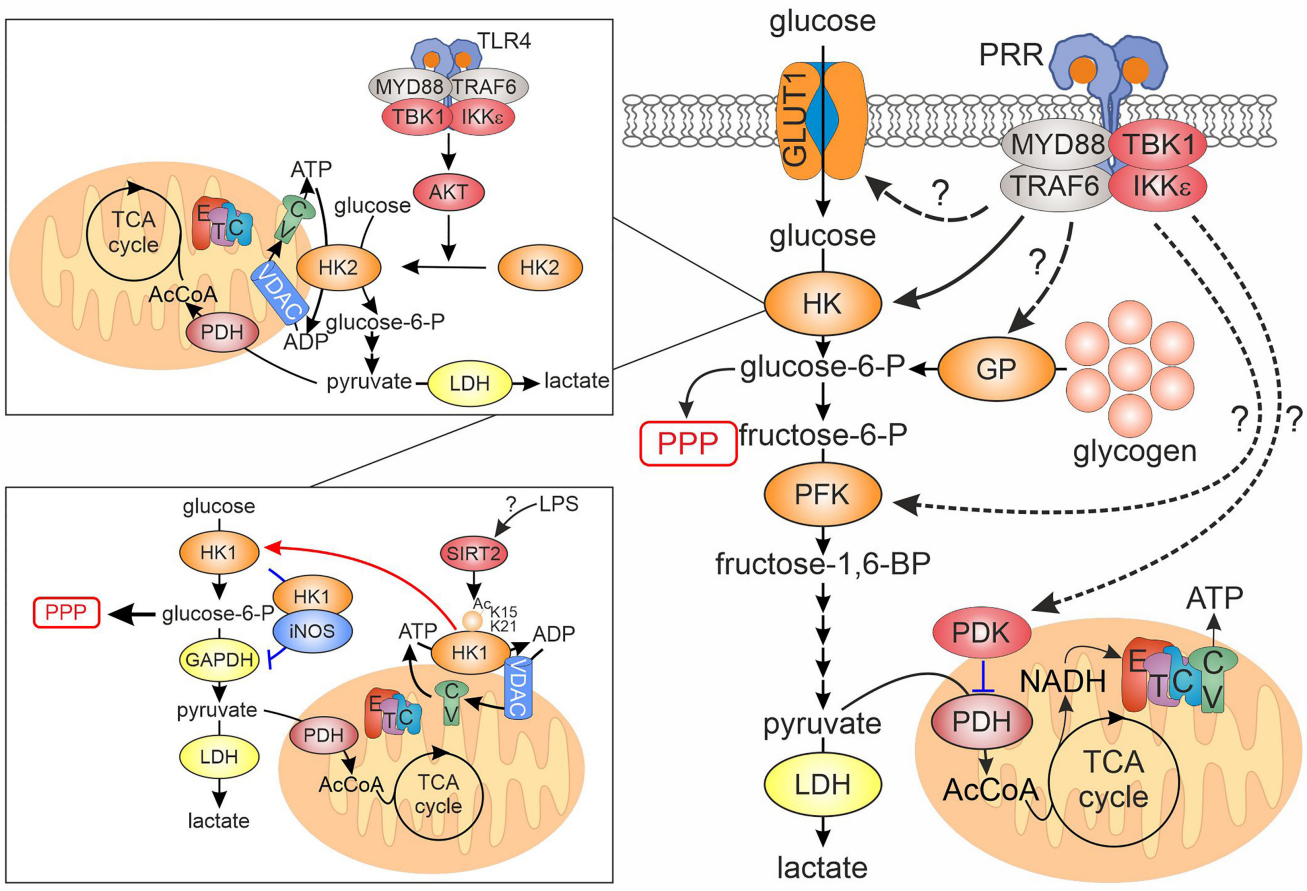
Regulation of rapid glycolytic activation upon PRR ligation in innate immune cells. PRR ligand recognition initiates the assembly of myddosome signaling complex, which, among others, includes the kinases TBK1 and IKKε. Activated myddosome is linked to the first step of the glycolytic cascade, hexokinase (HK), either through relocation of HK2 from the cytosol to mitochondria, or via movement of HK1 from mitochondria to cytosol (insets, details in the text). Other points of the glycolytic cascade affected by the activated myddosome include glucose entry via GLUT1 glucose transporter and glycogen breakdown via glycogen phosphorylase, but exact mechanistic details remain elusive. Whether regulation of the cascade via phosphofructokinase or pyruvate dehydrogenase kinase, reported for other cellular systems, is relevant for activated myeloid cells remains unknown. CV, complex V; ETC, electron transport chain; GAPDH, glyceraldehyde-3-phosphate dehydrogenase; GP, glycogen phosphorylase; HK, hexokinase; IKK, inhibitor of nuclear factor kappa B kinase; LDH, lactate dehydrogenase; MYD88, myeloid differentiation primary response gene 88; PDH, pyruvate dehydrogenase; PDK, pyruvate dehydrogenase kinase; PFK, phosphofructokinase; PPP, pentose phosphate pathway; PRR, pattern recognition receptor; TBK1, TANK-binding kinase 1; TCA, tricarboxylic acid; TLR, toll-like receptor; TRAF6, tumor necrosis factor receptor-associated factor 6; VDAC, voltage-dependent anion channel.

While an increased glycolytic metabolism upon innate immune activation was known for decades (13), the advent of real-time measurements of cellular metabolism allowed mechanistic investigations on the early signaling events accounting for an up-regulated glycolysis (14). First detailed investigation of a rapid glycolytic activation during the innate immune response was conducted in murine dendritic cells (DCs) (7). Extracellular flux analyses, i.e. real-time measurements of extracellular pH and oxygen consumption, showed that, upon toll-like receptor (TLR) ligation, DCs up-regulate extracellular acidification rates (ECAR) within 15-30 minutes, without changing oxygen consumption. Investigating 1,2-^13^C-glucose metabolism revealed increased pyruvate entry into the TCA cycle and lactate secretion. Mechanistically, the authors linked up-regulation of glycolysis to the activation of TANK-binding kinase 1 (TBK1)/IκB-kinase ε (IKKε) - Akt signaling in response to TLR ligation (**Figure 1**). Akt phosphorylated hexokinase (HK) 2, causing its translocation to mitochondria and increasing its activity. Consequently, a therapeutic peptide, promoting dissociation of HK2 from mitochondria, attenuated glycolytic activation by lipopolysaccharide. Glycolytic activation also occurred in DCs upon Dectin-1 receptor ligation (15). This depended on activity of a tyrosine kinase Syk, an early component of the Dectin-1 signal transduction cascade, as well as on the phosphatidylinositol 3-kinase/TBK1/Akt signaling. Interestingly, early glycolytic activation in DCs was induced even by stimuli causing weak inflammatory responses compared to LPS, such as house dust mite extract or depleted zymosan (16). Rapid glycolytic activation also occurs in human macrophages treated with LPS or with activators of cytosolic peptidoglycan receptors, nucleotide-binding oligomerization domain (NOD) 1 and NOD2 (17).

The involvement of TBK1 in glycolytic activation of macrophages was elaborated in a subsequent study (8). The authors showed that TBK1 is a component of a myddosome, a multiprotein signaling complex assembled around the adaptor protein MyD88 upon TLR activation. Following TLR ligation, TBK1 translocated to the myddosome via interaction with TNF receptor-associated factor 6 (TRAF6) where it was activated by phosphorylation. Pharmacological inhibition of TBK1/IKKε or silencing of TBK1 in IKKε-deficient bone marrow derived macrophages (BMDMs) prevented glycolytic activation without affecting other major branches of the TLR signaling cascade. This study did not address whether IKKε is also recruited to the myddosome. Thus, the relative contribution of the IKKε vs TBK1 to this signaling pathway remains unclear. Several independent studies confirmed that single deficiency of TBK1 or IKKε either does not affect glycolytic activation of macrophages (18) or has a partial effect (8). In contrast to short-term activation, neither TBK1/IKKε, nor Akt supported increased glucose consumption after 24-hour LPS stimulation in human DCs, which involved HIF1α-dependent transcriptional up-regulation of HK2 (19). Rather, p38 mitogen-activated protein kinase was involved.

Recent studies suggested additional mechanisms linking TLR signaling and the glycolytic cascade. Regarding the first intracellular step of the cascade, macrophages express three HK isoforms, HK1, HK2 and HK3 (20). HK1 is constitutively associated with mitochondria (21), where it is postulated to promote glycolysis, while a more labile HK2 was suggested to force glycogen synthesis and PPP in cytosol. HK3 does not localize to mitochondria, and its contribution to glycolysis may be limited (20). The role of HK1 mitochondrial location during the innate immune response was recently investigated (22). In contrast to observations in DCs, LPS induced mitochondrial dissociation of HK1, but not of HK2, in BMDMs. Mechanistically, the authors suggested that LPS induced sirtuin 2-mediated deacetylation of HK1 on lysines 15 and 21, promoting its release from mitochondria. How LPS elicits this deacetylation as well as the time scale of this process remains unclear. Using BMDMs from mice with a knockin of a mutant, exclusively cytosolic HK1, the authors observed the increased diversion of glucose towards the PPP upon LPS stimulation. At the same time, the flux of glucose towards pyruvate and lactate, and pyruvate entry into TCA cycle were decreased. The diversion of the glycolytic pathway occurred at the level of glyceraldehyde-3-phosphate dehydrogenase (GADPH). The authors suggested that cytosolic HK1 associated with S100 proteins S100A8/A9. This promoted inducible nitric oxide synthase (iNOS) -dependent S-nitrosation and inactivation of GADPH. Surprisingly, the authors detected levels of iNOS sufficient to nitrosate GAPDH in macrophages even under unstimulated conditions.

An additional pathway connecting TBK1/IKKε to glycolysis was suggested to involve phosphorylation of the transcription factor signal transducer and activator of transcription 3 (STAT3) at serine 727 (23). In response to TLR agonists in BMDMs, STAT3 translocated to the myddosome together with TBK1 via interaction with TRAF6. Here, STAT3 underwent phosphorylation at serine 727, but not at tyrosine 705, the canonical site of STAT3 phosphorylation in response to cytokine receptor stimulation. This phosphorylation was sensitive to TBK1/IKKε inhibition. As previously reported (24), serine 727-phosphorylated STAT3 accumulated in mitochondria. Peritoneal macrophages from mice with a knockin of a serine 727 to alanine-mutated STAT3 reduced both ECAR and oxygen consumption and attenuated an ECAR increase after LPS injection. The involvement of Akt or HK2 in STAT3 phosphorylation and mitochondrial translocation was not formally tested, but Akt inhibitors failed to suppress mitochondrial reactive oxygen species (ROS) generation, a readout of mitochondrial STAT3 activity.

Although most of the mechanistic research focused on the intracellular conversion of glucose to downstream metabolites, the entry of glucose into the cell may also undergo post-translational regulation. In murine macrophages, it is believed that most of glucose entry occurs via GLUT1 (SLC2A1) transporter (25, 26). While transcriptional regulation of GLUT1 expression during immune cell activation is quite established, GLUT1 may also undergo translocation from intracellular stores to the cell surface upon PRR activation, although with a delayed (2-12h) kinetics (27). Detailed mechanisms of this translocation remain unclear.

The glycolytic pathway is fueled not only through import and subsequent phosphorylation of glucose, but also through glucose-1-phosphate release from glycogen stores by glycogenolysis. The importance of this process in innate immune response was only recently appreciated (28). Murine DCs express both glycogen synthase and glycogen phosphorylase and, when cultured in glucose-containing media, build up glycogen stores. Intracellular glycogen is also present in human monocytes and DCs. Blocking glycogenolysis reduced both ECAR and oxygen consumption rates and lowered ATP levels in LPS-activated DCs. It also attenuated pyruvate entry into the TCA cycle. Inhibition of glycogen phosphorylase showed that glycogenolysis contributed to basal and LPS-stimulated glycolysis. Inhibition of glycogen phosphorylase also slowed glycolysis in DCs stimulated with TLR2 or Dectin agonists (29). Another study in BMDMs reported that both glycogen synthesis and glycogenolysis are up-regulated upon pro-inflammatory stimulation (30). Glycogenolysis-derived glucose-6-phosphate is predominantly channeled into the PPP. However, the overall contribution of glycogenolysis to glucose-6-phosphate levels in LPS/IFNγ-stimulated BMDMs was estimated to be less than 5% based on ^13^C-glucose tracer studies. Thus, the relative importance of glucose import vs glycogen breakdown during glycolytic activation of innate immune cells remains to be clarified.

Whereas a TBK1/IKKε-dependent glycolytic activation is an established mechanism for the innate immune response, another pathway was suggested to underlie glycolytic activation during the adaptive immune response (31). T cell receptor activation stimulated glycolysis in CD8^+^ T cells within minutes. Whereas no changes of phosphorylation of glycolysis-relevant enzymes were noticed during early activation, TCR ligation caused tyrosine phosphorylation of pyruvate dehydrogenase kinase 1 (PDK1). This activated PDK1 and increased inhibitory phosphorylation of pyruvate dehydrogenase. Inhibition of PDK1 with dichloroacetate or PDK1 silencing prevented initiation of glycolysis in T cells. PDK1 tyrosine phosphorylation was carried out by Zap-70 and Lck tyrosine kinases, which are part of classical TCR signaling cascade, whereas no involvement of Akt, mTOR, protein kinase C or calcium flux could be observed. It should also be noted that CD8^+^ T cells lack HK2, while mitochondria-localized HK1 appeared to be necessary to maintain respiration in activated memory CD8^+^ cells without effect on glycolysis (32). The impact of an inflammatory stimulus on PDH phosphorylation seems to be different in DCs, where reduced phosphorylation upon zymosan treatment was observed (33). In addition, only one of three inhibitory PDH phosphorylation sites (Ser300) increased in LPS-treated RAW264.7 macrophage-like cells, and rather an increased pyruvate to citrate flux was noticed (34). It should also be noted that mitochondrial respiration may also be activated by LPS under conditions of glucose restriction, suggesting metabolic flexibility during inflammatory responses (35, 36).

## 3 Early glycolytic activation supports pro-inflammatory responses

**Figure 2:**
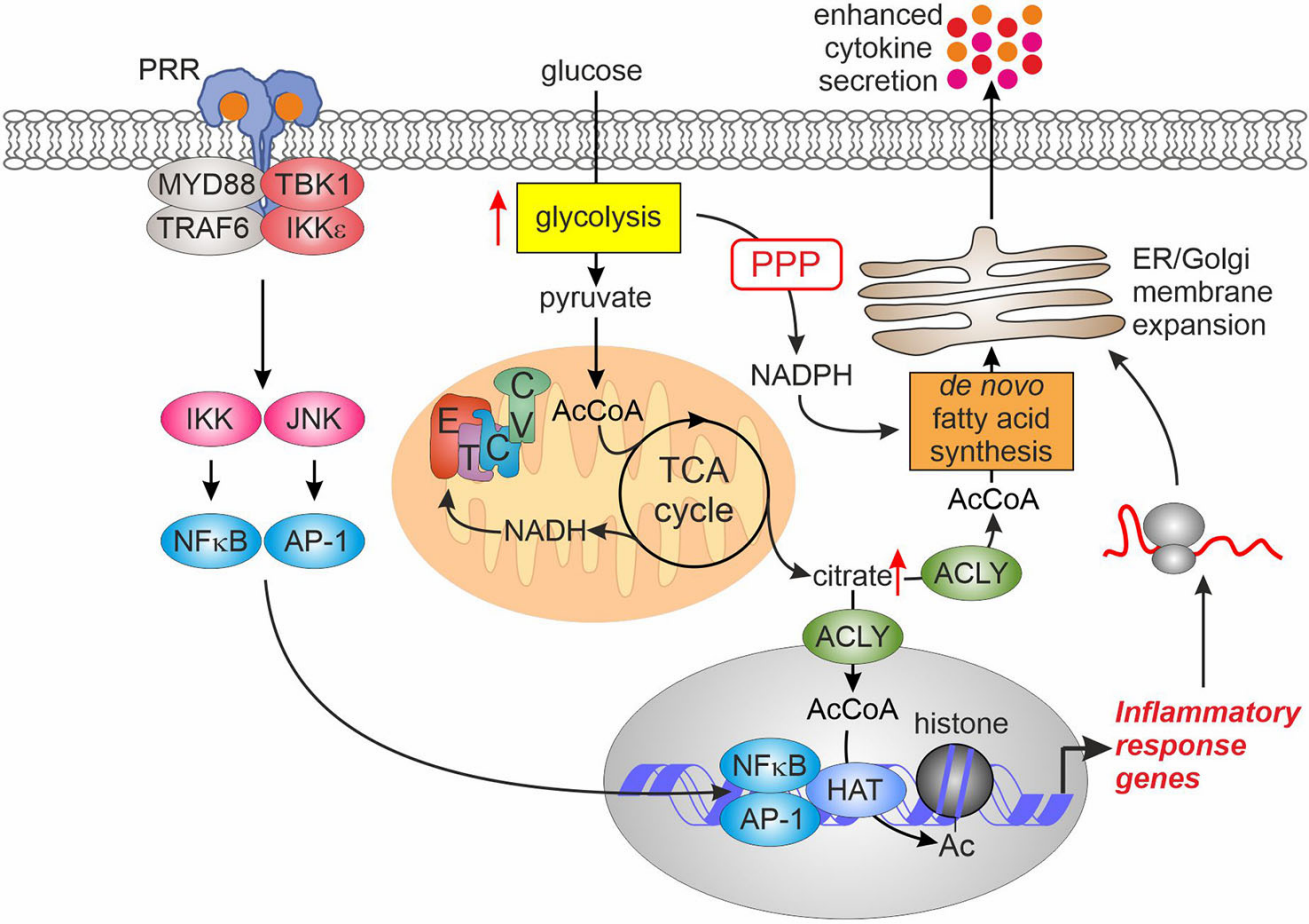
Glycolytic activation enhances inflammatory responses. Elevated glycolysis after PRR ligation enhances pyruvate entry into the TCA cycle. Consequently, this raises citrate export into cytosol where ACLY converts it to acetyl-CoA. Cytosolic acetyl-CoA feeds *de novo* lipogenesis to support expansion of ER and Golgi membranes necessary for enhanced cytokine secretion. In the nucleus, increased acetyl-CoA levels supply increased histone acetylation promoting transcription of inflammatory response genes. AcCoA, acetyl-CoA; ACLY, ATP-citrate lyase; AP-1, activator protein-1; CV, complex V; ER, endoplasmic reticulum; ETC, electron transport chain; HAT, histone acetyltransferase; IKK, inhibitor of nuclear factor kappa B kinase; JNK, c-Jun N-terminal kinase; MYD88, myeloid differentiation primary response gene 88; NFκB, nuclear factor kappa B; PPP, pentose phosphate pathway; PRR, pattern recognition receptor; TBK1, TANK-binding kinase 1; TCA, tricarboxylic acid; TRAF6, tumor necrosis factor receptor-associated factor 6.

The impact of metabolism on inflammatory responses of innate immune cells has been extensively investigated (see (2–4) for recent reviews). However, few studies focused on the initial phase, i.e. the first couple of hours post activation. Here, we summarize the recent findings on how early glycolytic activation may influence transcriptional, epigenetic, and post-transcriptional regulation of inflammatory responses (**Figure 2**).

A first study describing glycolytic activation of DCs also addressed the consequences of increased glycolysis (7). While glycolysis was dispensable for transcriptional responses to LPS in DCs (7), increasing pyruvate entry into the TCA cycle and resulting citrate generation provided a source for *de novo* fatty acid synthesis, necessary to support endoplasmic reticulum and Golgi complex membrane expansion. This, in turn, augmented protein biosynthetic and export capacity of DCs, resulting in elevated protein levels of cytokines and activation markers. In addition, increased activity of the PPP upon LPS-stimulation provided NADPH necessary to support fatty acid synthesis.

Augmenting the flux of pyruvate into the TCA cycle upon pro-inflammatory activation also evokes alterations of the macrophage epigenome linked to nucleocytosolic accumulation of acetyl-CoA derived from mitochondrial citrate. A recent study (37) investigating the response of BMDMs to LPS revealed accumulation of both, glycolytic and TCA cycle intermediates during the first 2 hours of activation. At the same time, LPS increased reductive TCA cycle flux of 13C-glutamine to citrate. LPS also induced Akt-dependent phosphorylation of ATP-citrate lyase (ACLY), an enzyme responsible for nucleocytosolic production of acetyl-CoA, causing its activation. The resulting accumulation of acetyl-CoA supported histone acetylation at promoters or enhancers of some LPS target genes characterized by a delayed response to LPS, such as interleukin 6 (IL6) or IL12B. Consequently, LPS-induced expression of these genes was also sensitive to inhibition of ACLY or mitochondrial pyruvate uptake. Similarly, ACLY was necessary to support LPS-induced histone acetylation and pro-inflammatory gene expression (exemplified by IL6 and IL1B) in another study (38). Here, the authors also observed increased incorporation of 13C-glucose in citrate and acetyl-CoA within 1.5-3 hours of LPS stimulation. Interestingly, this increase was dependent on the activity of mitochondrial glycerol-3-phosphate dehydrogenase (GPD2), a component of the glycerol-3-phosphate shuttle system. GPD2 deficiency attenuated glucose uptake of LPS-stimulated macrophages, 13C-glucose flux to citrate and acetyl-CoA, as well as LPS-stimulated promoter histone acetylation and mRNA expression of IL6 and IL1B. Mechanisms, how GPD2 supports oxidative metabolism of pro-inflammatory macrophages, remain to be clarified. Together, these studies suggest that increased glycolysis is primarily necessary to supply elevated citrate production in the TCA cycle to provide the substrate for nucleocytosolic acetyl-CoA synthesis. This in turn supports the increased demand for nuclear acetyl-CoA necessary for histone acetylation.

ACLY may not be the sole enzyme responsible for acetyl-CoA provision to support epigenome remodeling during inflammatory responses. Thus, increased histone acetylation at promoters of IL23 and IL10 sensitive to mitochondrial pyruvate carrier inhibition was noted in zymosan-treated human DCs, but inhibition of ACLY was without effect (33). Similarly, ACLY inhibition did not modulate histone acetylation or inflammatory gene expression in LPS-stimulated primary human macrophages (39). This necessitates further research to elucidate alternative sources of nuclear acetyl-CoA for histone acetylation at inflammatory stimulus-responsive genes.

In addition to the role of increased flux to pyruvate, enhanced PPP activity may also support inflammatory responses. Thus, BMDMs and peritoneal macrophages from cytosolic HK1-expressing mice displayed enhanced mRNA expression of IL1β, IL6, and TNFα upon LPS-stimulation, dependent on the PPP activity (22). The mechanistic details of this enhancement are unknown.

While most work on the early glycolytic activation followed the fate of pyruvate in mitochondria, the role of pyruvate to lactate conversion by lactate dehydrogenase (LDH), and lactate export during inflammatory responses, still requires detailed analyses. Silencing the lactate exporter monocarboxylate transporter 4 (MCT4) caused intracellular accumulation of lactate and attenuated inflammatory gene expression in response to TLR4 or TLR2 agonists in BMDMs (40). Increased cytosolic lactate after MCT4 depletion did not affect cytosolic inflammatory signaling cascades, suggesting transcriptional or epigenetic mechanisms. Intracellular lactate may attenuate inflammatory gene expression by regulating the expression of IRF4 transcription factor (41). Lactate may also covalently modify lysine residues on histone proteins (42), but this modification was rather associated with anti-inflammatory genes induced late in inflammatory response.

Several studies also addressed the impact of glucose entry via facilitative transporters, particularly, Glut1, on inflammatory responses in macrophages. Surprisingly, the data so far suggest that preventing glucose entry has only limited influence on inflammatory activation. Thus, human monocytes deprived of glucose show unaltered cytokine production and bacteria phagocytosis in response to LPS, the only functional impairment being attenuated ROS generation (36). Presumably, a shift of metabolism towards fatty acid oxidation upon glucose deprivation is sufficient to support metabolic requirements of inflammatory cytokine production. Considering Glut1 transporter, overexpression of Glut1 failed to augment inflammatory responses, but its deficiency attenuated inflammatory cytokine expression in LPS-stimulated BMDMs (43). Another study, however, reported no reduction in the secretion of IL6, IL1β or TNFα in LPS/interferon (IFN) γ -stimulated Glut1-deficient BMDMs (44). Here, compensatory increases of glutamine and fatty acid consumption in the TCA cycle were observed. Whether this flexibility in substrate use and corresponding adaptations of inflammatory responses also occur in the setting of acute inhibition of glucose entry remains to be established.

Most of the mechanistic work on the role of glycolysis was performed in cells stimulated by PRR ligands. An additional level of glycolysis regulation may be exerted by cytokine signaling. IFNγ (but not IFNβ) elicits glycolytic activation in BMDMs in an Akt-dependent manner similarly to PRRs (45), although such a response was not observed in human monocytes (46). Inhibiting LDH reduced IFNγ-induced cytokine production. Furthermore, granulocyte-macrophage colony stimulating factor (GM-CSF) augments LPS-induced glycolytic activation of BMDMs via up-regulation of glucose transporters in a Myc-dependent manner (47), and this was linked to enhanced inflammatory responses of GM-CSF-pretreated macrophages. On the other hand, IL10, an anti-inflammatory cytokine, suppresses glycolysis, while promoting oxidative phosphorylation in LPS-stimulated BMDMs (27). Mechanistically, IL10 prevents the translocation of GLUT1 to the cell surface upon LPS stimulation, and inhibits the expression of several enzymes of the glycolytic pathway.

## 4 Discussion

Recent research resulted in a significant gain of knowledge regarding mechanisms and functional roles of early glycolytic activation accompanying innate immune responses. Nevertheless, we still lack the detailed understanding of how TBK1/IKKε signals augment glycolysis, for example, whether these kinases have overlapping and/or redundant functions in the context of myddosome assembly and activation. The specific roles of individual hexokinase isoforms and their subcellular location upon activation should also be elucidated in future studies. Furthermore, the relative contribution of glucose entry vs glycogen store depletion to an increased glycolytic flux in different cellular systems needs clarification. Additional regulatory nodes, such as PFKFB3 or pyruvate dehydrogenase, merit consideration in future mechanistic studies. As these and other mechanistic details are investigated, more precise targeting of metabolic switches should also allow elucidation of the impact of glycolytic activation on transcriptional, epigenetic and post-translational responses to innate immune stimulation.

Most of the research to date focused on the innate immune responses elicited by the activation of PRRs. Thus, future areas of investigation should encompass a broader range of immune stimulatory or immunodulatory molecules, such as cytokines, G protein coupled receptor agonists, or growth factors, assessing their impact on the glycolytic activation.

We now appreciate that our understanding of immune cell metabolism may relate to quite a limited number of *in vitro* cellular models (mostly murine BMDMs or DCs), which cover only a part of possible metabolic phenotypes likely to occur in a living organism (4). Indeed, tissue-resident macrophages may have strikingly different metabolic patterns as compared to BMDMs. Thus, murine and human alveolar macrophages have low rates of glycolysis, which are not increased following LPS stimulation (48, 49). Consequently, alveolar macrophages are highly dependent on oxidative phosphorylation for the production of inflammatory mediators in response to LPS. Further mechanistic studies are thus warranted in these as well as other tissue-resident innate immune populations to understand relevant wiring of metabolic and signaling networks. Furthermore, differences in metabolism between murine and human macrophages should be taken into account, and remain largely unexplored (50). Development in this area may advance the application of metabolism-targeting drugs in infectious and inflammatory diseases.

## Author contributions

DN: writing the original draft. BB: editing the final draft. All authors contributed to the article and approved the submitted version.
